# The Plausibility of RNA-Templated Peptides: Simultaneous RNA Affinity for Adjacent Peptide Side Chains

**DOI:** 10.1007/s00239-012-9501-8

**Published:** 2012-04-27

**Authors:** Rebecca M. Turk-MacLeod, Deepa Puthenvedu, Irene Majerfeld, Michael Yarus

**Affiliations:** Department of Molecular, Cellular and Developmental Biology, University of Colorado, Boulder, CO 80309-0347 USA

**Keywords:** Aptamer, Binding, Histidine, Phenylalanine, Genetic code

## Abstract

According to the RNA world hypothesis, coded peptide synthesis (translation) must have been first catalyzed by RNAs. Here, we show that small RNA sequences can simultaneously bind the dissimilar amino acids His and Phe in peptide linkage. We used in vitro counterselection/selection to isolate a pool of RNAs that bind the dipeptide NH_2_-His-Phe-COOH with *K*_D_ ranging from 36 to 480 μM. These sites contact both side chains, usually including the protonated imidazole of His, but bind-free l-His and l-Phe with much lower, sometimes undetectable, affinities. The most frequent His–Phe sites do not usually contain previously isolated sites for individual amino acids, and are only ≈35 % larger than previously known separate His and Phe sites. Nonetheless, His–Phe sites appear enriched in His anticodons, as previous l-His sites also were. Accordingly, these data add to existing experimental evidence for a stereochemical genetic code. In these peptide sites, bound amino acids approach each other to a proximity that allows a covalent peptide linkage. Isolation of several RNAs embracing two amino acids with a linking peptide bond supports the idea that a direct-RNA-template could encode primordial peptides, though crucial experiments remain.

**Electronic supplementary material:**

The online version of this article (doi:10.1007/s00239-012-9501-8) contains supplementary material, which is available to authorized users.

## Introduction

Extant protein synthesis depends on the activity of proteins, but the initial encoded peptides must necessarily have been synthesized by non-protein agents. RNA is both capable of storing information and catalyzing reactions. Moreover, peptidyl transfer is today catalyzed by the RNA component of the ribosome (Nissen et al. 2000), and all other reactions of translation are arguably attainable using purified RNAs only (Yarus 2001). Therefore RNA is the most probable host for the first specific coded oligopeptide synthesis.

It has previously been shown (Zhang and Cech 1997; Illangasekare and Yarus 1999; Turk et al. 2010, 2011) that short peptides can be synthesized from activated amino acids (aminoacyl adenylates) in the presence of RNA. In addition, amino acids have been shown to polymerize on mineral surfaces (Lambert 2008). However, such peptides are not encoded; instead they arise from untemplated chemical polymerization.

An important question, then, is whether a specific sequence of amino acids can be directed by a particular RNA sequence, without intervention of proteins. The direct-RNA-template (DRT) hypothesis supposes that this can be accomplished by aligning activated amino acids in RNA aptamer sites, whose proximity facilitates peptide bond formation (Yarus 1998; Yarus et al. 2009).

Multiple RNA aptamers that bind eight different individual amino acids have been isolated (Yarus 1998) using in vitro selection (Tuerk and Gold 1990; Ellington and Szostak  1990; Joyce^ 1989^). In particular, aptamers for histidine (Majerfeld et al. 2005) and phenylalanine (Illangasekare and Yarus 2002) have been isolated and characterized, and found to bind their ligands with biochemically meaningful affinities and a high degree of specificity. Thus, we chose His–Phe as the target in this study because of previous characterization of the individual amino acid sites (reviewed in Yarus et al. 2009).

Nonetheless, it has been argued that peptide bond synthesis is impossible on a DRT, because two amino acids bound in adjacent RNA sites could not approach each other to the required bonding distance (Koonin and Novozhilov 2008; Ma 2010). Were this truly the case, then a DRT would be impossible. In that case, RNA adaptors (proto-tRNAs) may have been required, as well as, presumably, coding RNAs, from the primordial beginnings of translation. These latter ideas, however, are more complex than a DRT, because they require a mechanism to charge proto-tRNAs with particular amino acids.

We now support the simpler DRT option, showing that RNA sites directed to a peptide are not necessarily composed of adjacent amino acid aptamer sequences that must approach each other. Instead, we were able to isolate varied RNAs which selectively and simultaneously bind both side chains of dipeptide His–Phe in small sites. These findings support the DRT hypothesis by validating specific interactions between RNA and amino acids held at the proximity required for covalent peptide bonding.

## Materials and Methods

### Column Synthesis

To prepare His–Phe–Sepharose, EAH Sepharose 4B (100 μmol NH_2_) was dehydrated in *N*,*N*-dimethylformamide (DMF). 33 μmol Fmoc-Phe-OPfp (Novabiochem) in DMF and 66 μmol *N*,*N*-diisopropylethylamine (DIPEA) were added, and the solution was mixed for 120 min. Sepharose was washed with DMF, and the column was acetylated with 3 mmol acetic anhydride + 3 mmol DIPEA for 60 min. Fmoc was then removed by the addition of 20 % piperidine in DMF. Fmoc-His-Fmoc-OPfp (Bachem; 400 μmol in DMF) and 800 μmol DIPEA were then added, and the column was mixed for 4 h. The column was again acetylated, and Fmoc was removed by addition of 20 % piperidine in DMF. Concentration of His–Phe was determined by measuring OD_301_ of piperidine/DMF fractions to calculate the Fmoc eluted from the column. Final His–Phe concentration was 1.6 mM. This matrix was washed and stored in water.

### Library Synthesis

Single-stranded DNA was ordered from IDT. BR sequence: 5′-GGCAAGTACTCTGACGC(60N)GCGTAGAGGAGGACAGCG-3′. 3′-Primer sequence, 3′ DIP: 5′-CGCTGTCCTCCTCTACGC-3′. 5′-Primer sequence, T7 DIP: 5′-TAATACGACTCACTATAGGCAAGTACTCTGACGC-3′, where the underlined nucleotides indicate the T7 promoter.

Single-stranded DNA BR was converted to double-stranded DNA using PCR. PCR conditions: 1×* Taq* buffer (Promega), 0.41 mM dNTPs, 4.4 mM MgCl_2_, 4.5 μM each primer, 0.45 μM BR DNA, 0.046 units/μl* Taq* polymerase (Promega). Cycles: 94 °C for 5 min; *94 °C for 30 s, 50 °C for 30 s, 72 °C for 2 min; repeat from “*” 5 times; 72 °C for 7 min. Amplified DNA was ethanol-precipitated and resuspended in water.

RNA was transcribed from dsDNA as follows: 1× T7 Flash buffer (Epicentre), 7.5 mM NTPs, 10 mM DTT, 0.125–0.25 μM (1.5 mCi/ml) α-^32^P-GTP or CTP, 1–10 μM DNA, 1× T7 Flash enzyme (Epicentre). Incubation was at 37 °C overnight. Transcription was then treated with DNase I (Epicentre), gel purified, and ethanol-precipitated.

### Selection

Before column affinity chromatography, ^32^P-labeled RNA was folded by heating at 65 °C for 3 min, adding 5× column buffer to a final concentration of 1× (50 mM HEPES pH 7.0, 600 mM NaCl, 5 mM MgCl_2_, 5 mM CaCl_2_), and reheating for 30 s. Each cycle (except for Cycle 1) was preceded by a counter-selection against RNAs that nonspecifically bound to acetylated Sepharose. RNA (1 nmol for cycle 1) was then applied to His–Phe–Sepharose columns (equilibrated with 8 column volumes column buffer) and eluted with 1.5 mM His–Phe in column buffer (Cycle 1), or 1.5 mM His, 1.5 mM Phe, and then 1.5 mM His–Phe in column buffer (subsequent cycles). Columns were then washed with high salt buffer: 50 mM HEPES pH 7.0, 1 M NaCl, 2 mM EDTA. Desired fractions were pooled, precipitated, and reverse-transcribed into DNA using AMV reverse transcriptase (Life Sciences, Inc.). ssDNA was amplified by PCR (as for library amplification), and the cycles were repeated until a significant fraction of the RNA (by scintillation counts) was shown to elute from His–Phe columns upon addition of His–Phe. This RNA was cloned (Novagen PT7 Blue-3 Perfectly Blunt Cloning Kit) and sequenced by Sanger methods.

### Chemical Probing

RNA was folded as previously but with the addition of 5.5× SHAPE folding buffer to 1× (final 50 mM HEPES pH 7.5, 400 mM NaCl, 5 mM MgCl_2_, 5 mM CaCl_2_). Modification of RNA by *N*-methylisatoic anhydride (NMIA) was carried out as in (Wilkinson et al. 2006), using 13 mM NMIA and incubating at 24 °C for 155 min, or 37 °C for 45 min. Protection experiments used ~1 pmol RNA; RNA, His–Phe, and NMIA were incubated as in modification experiments. For binding interference/enhancement experiments, ^32^P-labeled RNA was NMIA-modified, passed through a Micro Bio-Spin 6 chromatography column (Bio-Rad) exchanged with column buffer, and subjected to affinity chromatography through a His–Phe–Sepharose column. Unbound fractions followed by fractions eluted upon addition of 1.5 mM His–Phe were collected, ethanol-precipitated, and reverse-transcribed for PAGE analysis. PAGE used 8 % acrylamide/7 M urea in TBE, run at 1,600 V until xylene cyanol had migrated 16.5 cm.

## Results

### Selection for His–Phe-Binding RNA

**Fig. 1:**
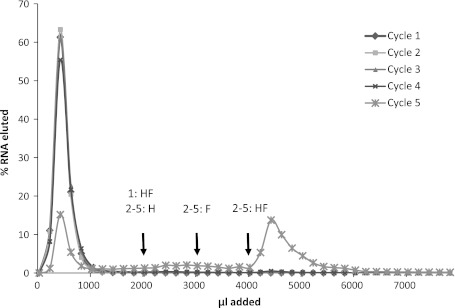
Elution profiles of RNA from His–Phe column during selection. 1.5 mM His (H), Phe (F), or His–Phe (HF) in column buffer was applied to His–Phe–Sepharose column as indicated. *Numbers* indicate the cycle in which the eluant was applied. Only fractions collected upon addition of His–Phe were pooled for further selection

His–Phe-binding RNAs were isolated via in vitro selection using RNA affinity chromatography. ^32^P-labeled RNA with a tract of 60 contiguous randomized nucleotides flanked by constant primer-complementary sequences was applied to columns of His–Phe–Sepharose. Initial selection experiments showed that straightforward selection for dipeptide affinity frequently yields individual amino acid aptamers which do not detectably bind the second amino acid. Thus, the revised selection goal was to remove strongly directed His and Phe sites from the randomized RNA pool, leaving RNAs which would bind His–Phe with substantial affinity, and possibly His and Phe, but only with lower affinity. Thus, every selection cycle (with the exception of the first) was preceded by a counterselection which discarded RNAs eluted by free l-His and l-Phe. RNA eluted upon subsequent addition of 1.5 mM His–Phe peptide was pooled, reverse-transcribed, PCR-amplified, and transcribed back into RNA for re-selection. In the fifth cycle, 50 % of RNA was eluted by addition of peptide to the His–Phe–Sepharose (Fig. 1).

**Fig. 2:**
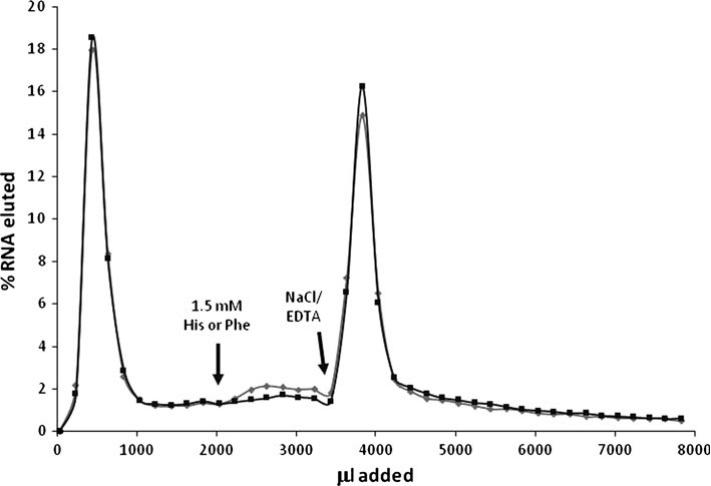
Pool RNA elution profiles from His–Phe columns upon addition of free His and Phe. 1.5 mM His (*diamonds*) or 1.5 mM Phe (*squares*), and 1 M NaCl/2 M EDTA/50 mM HEPES pH 7.0 were added as indicated

**Fig. 3:**
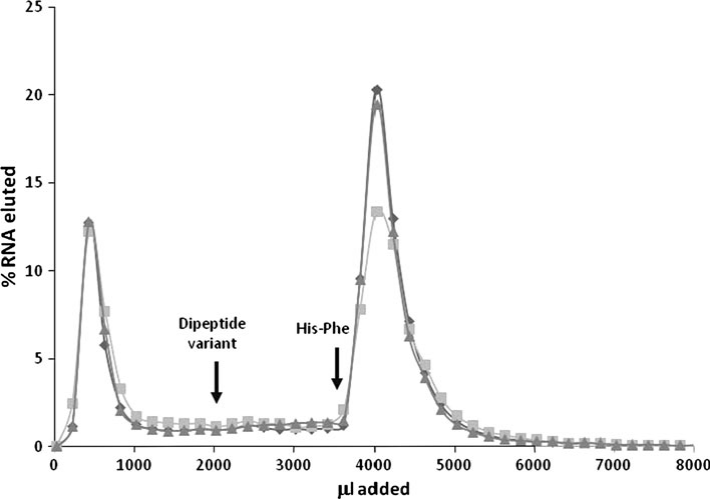
Selected RNA specifically binds the dipeptide His–Phe. 1.5 mM dipeptide variant and His–Phe were added to His–Phe–Sepharose columns as indicated. Dipeptide variants: *diamonds* Gly–Phe, *squares* His–Gly, *triangles* Phe–His-OCH_3_

In this selected RNA population, free amino acid affinities were usually small. Upon addition of histidine or phenylalanine individually, selected RNAs eluted feebly, if at all, from the His–Phe column (Fig. 2). Therefore, the strategy of discarding strongly eluted His- and Phe-binding RNAs appears effective. Elution of pooled selected RNAs was also tested using congeners of the His–Phe peptide. His–Gly, Gly–Phe, and Phe–His methyl ester (Phe–His-OCH_3_) did not elute selected RNA (Fig. 3). Thus, RNA affinity is not generally for peptides, but specifically for the selective His–Phe peptide.

### Clone Sequence and Binding Characteristics

**Fig. 4:**
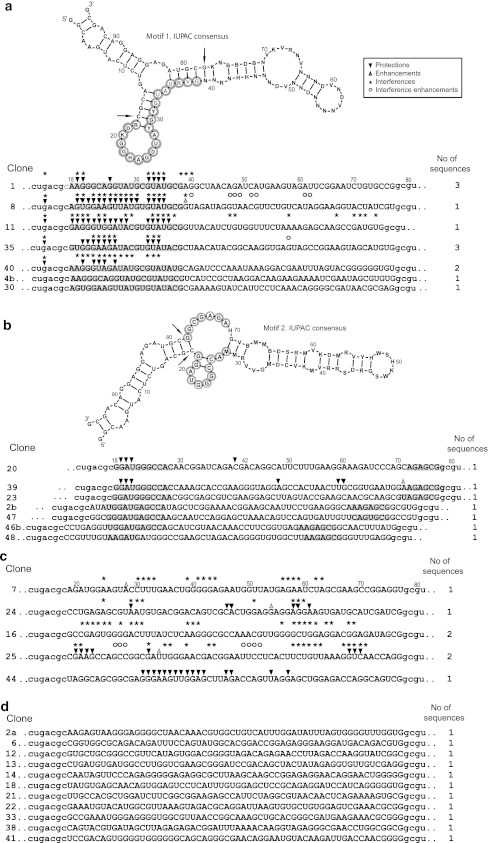
Cloned sequences from selection. The secondary structure heading of the first two panels is the most stable for multiple occurrences of the conserved motif, as estimated by Bayesfold (Knight et al. 2004), shown with the IUPAC consensus for the motif threaded through the stable structure. *Arrows* point to last primer nucleotides. IUPAC symbols used are: *N* A/C/G/U, *R* G/A, *Y* C/U, *M* A/C, *K* G/U, *W* A/U, *S* C/G, *B* not A, *D* not C, *H* not G, *V* not U. **a** Initial variable region of cloned sequences aligned to display the major conserved motif, isolated seven times independently (nt with *gray background*). *Symbols* above the sequence summarize differences in chemical susceptibility to NMIA ± HF peptide. **b** The second frequent motif aligned and threaded through its estimated most stable common structure. These conservations fold back to jointly form a conserved loop system, isolated seven times independently (nt with *gray background*). *Symbols* above the sequence summarize differences in chemical susceptibility ± HF peptide. **c** Chemical data for uniquely isolated sequences which were tested for chemically implicated nucleotides. **d** Other sequences not tested for implicated nucleotides

Cycle 5 RNA was reverse-transcribed to DNA, PCR-amplified, cloned, and sequenced. Alignment reveals a common motif in 7/30 clones RDKGGHAGDUAYGYGUAURYG (Motif 1, Fig. 4a). This repetitively isolated sequence is notable because this motif presents a high frequency of RUG His anticodons (Yarus et al. 2009; Illangasekare et al. 2010), as indeed do RNA sites for free His itself (Majerfeld et al. 2005). However, none of the cloned RNAs appears to contain the entire, repetitively isolated previous histidine-binding site (Majerfeld et al. 2005; Illangasekare et al. 2010), consistent with the observation that present RNAs do not usually bind-free histidine with high affinity (Fig. 2). Sequences from the most frequently observed Phe-binding sites (Illangasekare and Yarus 2002) were also not present in the peptide-binding RNA population. A single exceptional sequence, RNA 44, binds phenylalanine as well as His–Phe, but does not bind histidine (Supp. Fig. 1). This characteristic was likely invisible in the column chromatography analysis of pool RNAs (Fig. 2), due to the relatively low proportion of pooled RNA 44 sequences (Fig. 4), and is not attributable to recurrence of a salient, previously isolated (Illangasekare and Yarus 2002) Phe site.

**Fig. 5:**
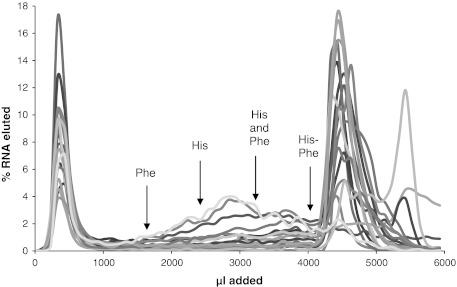
Binding characteristics of clones compared. Superposed, independent elution profiles for RNAs 1, 6, 7, 8, 11, 12, 13, 16, 20, 22, 23, 24, 25, 33, 35, 39, 40, and 48 from a His–Phe affinity column. 1.5 mM His, Phe, His and Phe, or His–Phe dipeptide in column buffer were added as indicated

None of the eighteen other selected RNAs was definitely eluted by His, Phe, or even by equimolar-free His and Phe simultaneously (as measured by elution from a His–Phe peptide column) though all the eighteen bound His–Phe dipeptide (Fig. 5). This does not exclude the possibility, however, that the RNAs might bind the individual amino acids with relatively low affinity (too low to detectably compete against His–Phe–Sepharose). Therefore, several clones with substantial His–Phe peptide affinity were chosen for individual measurement of *K*_D_ for His, Phe, His–Phe, and the related dipeptides His–Gly and Gly–Phe.

*K*_D_ was determined by isocratic elution from His–Phe–Sepharose affinity columns as in Ciesiolka et al. (1996) (Table 1). RNAs 16, 25, and 40 have *K*_D_ less than 100 μM for His–Phe; these values are comparable to, e.g., *K*_D_ previously measured for His aptamers, 8–54 μM (Majerfeld et al. 2005).

**Table 1:** Dissociation constants( *K*_D_) measured for His–Phe aptamers by affinity elution at pH 7.0

	His–Phe (μM)	His (mM)	Phe (mM)	His–Gly (mM)	Gly–Phe (mM)
RNA 7	478 ± 61	>119	22	10.5	31.6
RNA 8	102 ± 33	>34	>34	5.4	>34
RNA 16	87 ± 3	13	100	17	>587
RNA 25	36 ± 2	>727	>727	8.2	>727
RNA 40	77 ± 7	>227	>227	3.4	9.3

*K*_D_ (±SE of the mean) are shown for specified ligands

Even though the addition of His and Phe individually, or together, did not produce a definite elution peak for most clones (Fig. 5), analysis of *K*_D_ in the presence of high concentrations of His and Phe and/or related dipeptides is more resolving, offering insight into weaker affinities of the His–Phe aptamers. For example, Table 1 shows that RNA 16 is unusual in showing measurable, low affinities for His and Phe individually, though ≈two to three orders of magnitude weaker than for dipeptide His–Phe. Presumably, increased molecular interaction with sites on His–Phe peptide ligand is decisive in this case.

Peptide bond atoms themselves can interact with the RNA sites. All sequences tested have low but detectable affinity for other peptides, His–Gly and/or Gly–Phe. In cases where this *K*_D_ is lower than that of free His or Phe, peptide bond atom groupings plausibly interact with the RNA-binding site (Table 1).

**Fig. 6:**
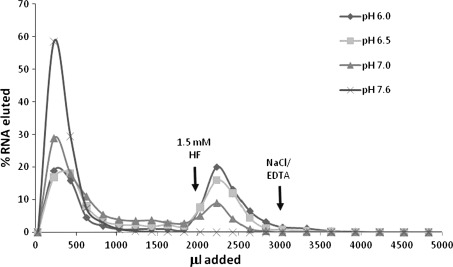
Elution profiles of RNA 8 at varied pH

Affinity for His–Phe often includes affinity for a protonated imidazole side chain. Three sequences were tested for His–Phe binding under slightly more acid conditions (0.1 M HEPES, pH 6.0). All the three RNAs showed an increased affinity for His–Phe, with *K*_D_ as low as 8 % of the measured *K*_D_ at pH 7.0 (Fig. 6; Table 2). Further, RNA 8 shows a regular progression of decreasing His–Phe column affinity with increasing pH (Fig. 6). These data suggest that the protonated imidazole of the His side chain is an RNA-bound ligand. This increased affinity may be as simple as coulombic interaction with the protonated imidazole, but a characteristic pH dependence implicates the His side chain in the binding interface.

**Table 2:** His–Phe *K*_D_ for selected clones at pH 6.0

	*K*_D_ His–Phe (μM)	% of *K*_D_ at pH 7.0
RNA 8	41 ± 2	40
RNA 16	8 ± 1	9
RNA 40	22 ± 0.4	29

### Structural and Functional Analysis of Selected Clones

Chemical probing using *N*-methylisatoic anhydride (NMIA), which reacts with ribose 2′-OH of accessible nucleotides (Wilkinson et al. 2006), was performed for RNAs 1, 7, 8, 11, 16, 20, 24, 25, 35, 39, 40, and 44 (Fig. 4). Clones were chosen based on substantial affinity for His–Phe (measured by column affinity chromatography) and/or because they differ in sequence sufficiently to represent independent isolations (originating in different initial molecules) of frequent sequence motifs.

Protection and binding enhancement/interference experiments revealed nucleotides important for binding. In particular, ribose protected from modification when incubated with His–Phe, or ribose molecules which, once modified, prevent binding to His–Phe probably interact with peptide, are adjacent to such site nucleotides or linked to them within a folding domain.

There are multiple RNA folds that bind His–Phe. Two-dimensional structure prediction using BayesFold (Knight et al. 2004) and protection or binding interference/enhancement after chemical probing indicate two recurring motifs in the RNA random region which are likely important for binding to His–Phe (Fig. 4). The RDKGGHAGDUAYGYGUAURYG motif (Motif 1) appears in seven-independent cases (five have been characterized chemically) and often frames a hairpin loop and helix junction. Notably, that motif is not found at all in RNAs 16 and 25, suggesting that there exist multiple easily isolated His–Phe-binding RNA structures. In agreement with this expectation, a second complex internal loop site (Motif 2) is nearly equally frequent, with seven-independent isolations (Fig. 4) of the GGAUGGGCC…AGAGCGG sequence. In this case, conserved nucleotides are in two tracts which loop back to fold together. Motif (recurring) nucleotides are also implicated by NMIA chemical data (two-independent cases), as for the first motif. Among other structures, the linear structure of RNA 16 predicts binding site nucleotides in two widely separated regions (Fig. 4) of a novel sequence, intriguing because of the two underlying clusters of ligand atoms. Taken together, observed variability in sequence and structure suggests independent, simple molecular routes to His–Phe affinity in RNA.

To objectively discuss the size of amino acid and peptide sites, we define an “implicated site nucleotide” (ISN) as any selected nucleotide that is conserved in independent isolates, or implicated in binding by chemical criteria (e.g., protection, sensitization, interference), or both conserved and chemically implicated. The sum of these “implicated nucleotides” therefore counts every nucleotide whose function is supported by recurrence, chemical experiment or both. The count of implicated nucleotides in His, Phe, and His–Phe sites is compared in Table 3. From these data, it appears that the most frequently isolated (simplest) dipeptide sites are not the size of summed amino acid sites, but instead have only 30–40 % more nucleotides than single His and Phe sites. This reinforces the conclusion drawn above from the absence of the predominant His- and Phe-binding sequences in the peptide-binding population: the simplest peptide sites are unique chemical entities, not composed by assembling the likely sites for free amino acids. Further, the fractional increase in implicated nucleotides in the dipeptide site is more consistent with a “side-by-side” (and therefore overlapping) orientation of the two amino acids than an extended orientation in which bound peptide amino acids would present near-total interaction surfaces to a surrounding RNA. Such relative positioning, though the argument is indirect, is consistent with the requirements of a DRT mechanism (Yarus et al. 2009).

**Table 3:** Randomized nucleotides (Random) and implicated site nucleotides (ISN) in the binding sites for independently isolated His sites (Majerfeld et al. 2005), Phe sites (Illangasekare and Yarus 2002), and His–Phe sites (this study, cf. Fig. 4)

	His random	His ISN	Phe random	Phe ISN	His–Phe random	His–Phe ISN
Nucleotides	70	20.1	80	17.5	60	24.4 ± 1.7

The number of implicated nucleotides is the average of 13 independently selected sites (His), two-independent sites (Phe) or seven independently isolated sites of two types (His–Phe; mean ± SEM is shown) for which both conserved nucleotides and chemical data were measured

A modular DRT, with independent amino acid sites brought together by a linking sequence, is not ruled out by our results, but Table 3 suggests that such a structure would require many more specified nucleotides than our selected sites. That is, two complete amino acid sites, as well as a linking sequence which allows the sites to approach for peptide formation, would be required. Therefore such structures would likely be much less frequent than the smaller peptide sites which dominate our selections.

## Discussion

We have selected different, independent RNA sequences that bind His–Phe. Strongly implicated peptide-binding nucleotides may either be in one contiguous region (RNA 8) or possibly separated into two tracts (RNA 16, RNA 20, Fig. 4). Even RNAs with similar *K*_D_ have different sequences, and also have apparently different secondary structures and possible site motifs. Varying RNA affinities for the individual amino acids as well as related dipeptides indicate that His–Phe aptamers may bind different parts of the His–Phe dipeptide with high affinity, though none characterized here appears to concentrate on one amino acid exclusively. RNA 16, in particular, has measurable affinity for both the individual amino acids as well as for the dipeptide. It therefore meets the most elementary requirements of a DRT, which must bind and order activated, subsequently reacting, amino acid substrates (Yarus 1998; Yarus et al. 2009).

These data bear on alternative affinity, a significant independent explanation for affinity for both His and Phe. An alternatively bound peptide would behave as follows:

$$ {\text{RNA}}^{{{\text{H}} - {\text{F}}}} \leftrightarrow {\text{RNA}}_{{{\text{F}} - {\text{H}}}} $$

with peptide H–F being bound via either the His moiety (RNA^H–F^) or the Phe moiety (RNA_F–H_). Such an equilibrium permits His plus Phe affinity without simultaneous binding, and would be thereby unusable for a DRT. However, present RNAs infrequently show reduced affinities for dipeptide in the presence of either His or Phe (Table 1). For equally divided Δ*G*_binding_, *K*_binding_ would decrease to *(K*_binding_)^½^ in the presence of high concentrations of either amino acid; e.g., from 100 μM to 10 mM. Most tellingly, even for the one case where affinity for both His and Phe is detectable (RNA 16, Table 1), when both free amino acids are present at once no RNAs are strongly eluted, eliminating the alternate-binding model above (Fig. 5). Therefore alternative His and Phe binding is not a frequent route to His–Phe affinity among our peptide-binding RNAs. Such sites might still exist, perhaps reduced or discarded by our counterselection, but clearly, multiple peptide-binding RNAs have been isolated that do not show a characteristic sensitivity to alternately bound amino acids (Table 1; Fig. 5).

The five RNAs of Table 1 discriminate both amino acid side chains in His–Phe and similar peptides. It seems likely, then, that it is relatively easy for an RNA site to bring two amino acids together in proximity, likely side-by-side and close enough for peptide bond formation to occur. Our results suggest that the simplest way to do this is to construct a side-by-side site for the two amino acid side chains, but our results cannot rule out more complex and therefore rarer, molecular strategies.

The DRT mechanism can be compared to the ribosome, which functions to position tRNAs and their respective amino acids in a conformation favorable for peptidyl transfer (Sievers et al. 2004). Since the 2′-OH of the aminoacyl substrate may catalyze extant peptide bond formation (Weinger et al. 2004), it would be plausible to conduct peptidyl transferase experiments for these RNAs using His–Phe aptamers and amino acids activated as esters, particularly as esters of ribose or nucleotides.

**Fig. 7:**
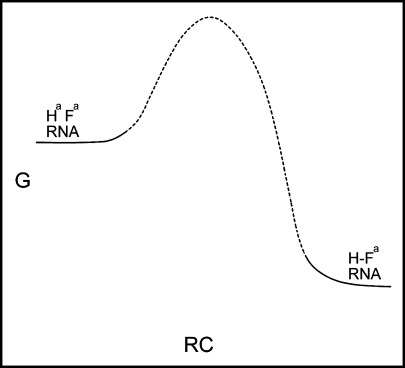
Possible reaction co-ordinate for a peptide aptamer which is also a peptidyl transferase.* Lower case* *a* represents a hypothetical activating group, required for peptide formation but not studied in these experiments. See "Discussion" section for other comments on relation to RNAs selected here, where only states related to the *right* (peptide product) and *left* (activated amino acids) have been directly observed

Peptide synthesis experiments suppose that these peptide-binding sites might template peptide formation. This requires comment on the distance between present observations and observable DRT action. In these experiments, only weak substrate (amino acid) and product (peptide) affinity have been demonstrated (e.g., for RNA 16, Table 1). But there is potential for peptide synthesis related to the Hammond postulate (Fersht 1999). In generalized form, the Hammond postulate asserts that nearby points on the free energy progress diagram for a reaction are similar structurally. Here, we select affinity for peptide and (due to counterselection) weaker affinity for amino acids. Weak amino acid affinity is significant because it may be useful in shortening the path to the transition state. Some selected RNA structures may therefore allow weakly bound ester substrates to progress through a nearby transition state for peptide formation (Fig. 7). The crucial transition state may be structurally bracketed, in the Hammond sense, between bound amino acid substrates and peptide products. Peptide products are more stable, and would accumulate (Table 1). It remains to be seen whether or not these nine His–Phe aptamers allow access to the peptide bond-forming transition state under some accessible and biochemically plausible solution condition. But, even if this initial population is not catalytic, catalytic Hammond variants may exist in sequence space (for example, near RNA 16. cf Table 1), thus completing the repertoire of the DRT.

These results also bear on the DRT hypothesis itself, which posits that modern coding triplets were originally sequences within cognate ribo-oligomer amino acid binding sites (Yarus 1998). His–Phe aptamers do not contain an excess of Phe codons or anticodons, consistent with previous Phe aptamers and with the hypothesis that the genetic code for Phe may have evolved later in evolution (Illangasekare and Yarus 2002). However, these His–Phe sites do contain an elevated frequency of RUG His anticodons, in the following sense. Taking together the two most frequent motifs for His–Phe binding (Fig. 4a, b), there are 12 RUG sequences at 157 possible triplet sites (counting only sites not constrained by base-pairing with the supplied terminal constant sequences). This is a frequency of 0.076. Because RUG sequences are expected in random-sequence oligonucleotides at only (2/64=) 0.031 of possible triplets, ≥0.076 RUG would be expected only ≈4 × 10^−3^ of the time (binomial expectation of ≥0.076 if 0.031 is the true frequency). RUG anticodons for His accordingly recur improbably frequently within His–Phe-binding sites. Because we have shown that the most prevalent His–Phe peptide-binding sites are not composed of the most prevalent amino acid sites, this apparent His anticodon excess occurs in a new class of RNA-amino acid interactions. These data therefore present a new level of evidence consistent with a DRT, and thereby, consistent with a stereochemical explanation of the genetic code for His (Yarus et al. 2009; Majerfeld et al. 2005; Illangasekare et al. 2010).

## Electronic supplementary material

Below is the link to the electronic supplementary material.

Supplementary material 1 (PDF 224 kb)
