# Supplementary material for: The Plausibility of RNA-Templated Peptides: Simultaneous RNA Affinity for Adjacent Peptide Side Chains
Source: J Mol Evol. 2012 Apr 27;74(3):217–25. doi: 10.1007/s00239-012-9501-8 (PMC3346935; doi:10.1007/s00239-012-9501-8)
Supplement: Supplementary file 1 — Supplementary material 1 (PDF 224 kb) [file 239_2012_9501_MOESM1_ESM.pdf]

Simultaneous RNA affinity for adjacent peptide amino acids
Journal of Molecular Evolution
Rebecca M. Turk-MacLeod, Deepa Puthenvedu, Irene Majerfeld, and Michael Yarus
Department of Molecular, Cellular and Developmental Biology
University of Colorado
yarus@stripe.colorado.edu

a

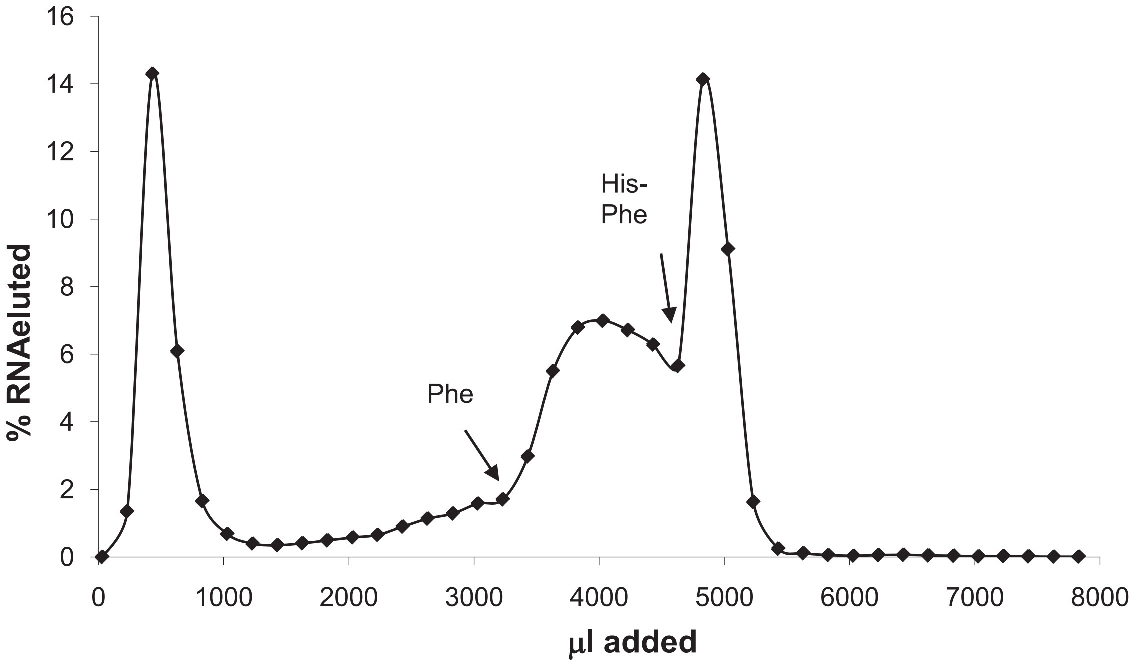

b

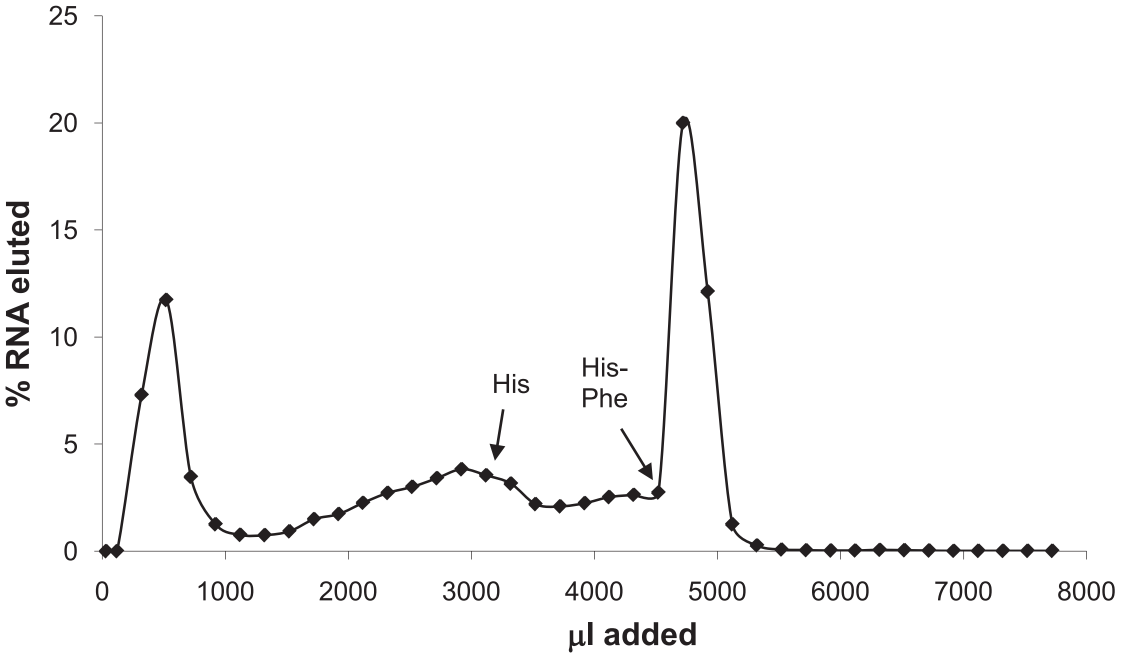

Fig. S1. RNA 44 elutes from His-Phe column upon addition of Phe, but not His. a: Phe elution. **b**: His elution. 1.5 mM His, Phe, or His-Phe in column buffer were applied to the column as indicated.
